# Yeast-model-based study identified myosin- and calcium-dependent calmodulin signalling as a potential target for drug intervention in chorea-acanthocytosis

**DOI:** 10.1242/dmm.036830

**Published:** 2019-01-28

**Authors:** Piotr Soczewka, Damian Kolakowski, Iwona Smaczynska-de Rooij, Weronika Rzepnikowska, Kathryn R. Ayscough, Joanna Kaminska, Teresa Zoladek

**Affiliations:** 1 Institute of Biochemistry and Biophysics, Polish Academy of Sciences, Department of Genetics, Pawinskiego 5A, 02106 Warsaw, Poland; 2 Department of Biomedical Science, University of Sheffield, Sheffield S10 2TN, UK

**Keywords:** Yeast, Chorea-acanthocytosis, Vps13, Myo3, Calcium signalling, Endocytosis

## Abstract

Chorea-acanthocytosis (ChAc) is a rare neurodegenerative disease associated with mutations in the human *VPS13A* gene. The mechanism of ChAc pathogenesis is unclear. A simple yeast model was used to investigate the function of the single yeast VSP13 orthologue, Vps13. Vps13, like human VPS13A, is involved in vesicular protein transport, actin cytoskeleton organisation and phospholipid metabolism. A newly identified phenotype of the *vps13*Δ mutant, sodium dodecyl sulphate (SDS) hypersensitivity, was used to screen a yeast genomic library for multicopy suppressors. A fragment of the *MYO3* gene, encoding Myo3-N (the N-terminal part of myosin, a protein involved in the actin cytoskeleton and in endocytosis), was isolated. Myo3-N protein contains a motor head domain and a linker. The linker contains IQ motifs that mediate the binding of calmodulin, a negative regulator of myosin function. Amino acid substitutions that disrupt the interaction of Myo3-N with calmodulin resulted in the loss of *vps13*Δ suppression. Production of Myo3-N downregulated the activity of calcineurin, a protein phosphatase regulated by calmodulin, and alleviated some defects in early endocytosis events. Importantly, ethylene glycol tetraacetic acid (EGTA), which sequesters calcium and thus downregulates calmodulin and calcineurin, was a potent suppressor of *vps13*Δ. We propose that Myo3-N acts by sequestering calmodulin, downregulating calcineurin and increasing activity of Myo3, which is involved in endocytosis and, together with Osh2/3 proteins, functions in endoplasmic reticulum-plasma membrane contact sites. These results show that defects associated with *vps13*Δ could be overcome, and point to a functional connection between Vps13 and calcium signalling as a possible target for chemical intervention in ChAc. Yeast ChAc models may uncover the underlying pathological mechanisms, and may also serve as a platform for drug testing.

This article has an associated First Person interview with the first author of the paper.

## Abstract (precis)

**Summary:** Using the *vps13*Δ strain, a yeast model of the neurodegenerative disorder chorea-acanthocytosis, we found that its defects can be overcome by reduction of calcineurin activity and/or type-I-myosin activation.

## INTRODUCTION

The VPS13 (vacuolar protein sorting 13) proteins are conserved among eukaryota. In humans, the VPS13 protein family consists of four members encoded by the *VPS13A*, *B*, *C* and *D* genes (Velayos-Baeza et al., 2004). Mutations in *VPS13A* lead to a rare, fatal neurodegenerative disease: chorea-acanthocytosis (ChAc; OMIM 200150) (Rampoldi et al., 2001; Rubio et al., 1997; Ueno et al., 2001). ChAc is characterised by many neurological symptoms, including: chorea; dystonia and twitches; and, often, the presence of acanthocytes (erythrocytes with a spiked morphology) (Hardie et al., 1991). Several studies have shown a role of the VPS13 proteins in cytoskeletal organisation (De Franceschi et al., 2011; Föller et al., 2012), vesicular transport (Honisch et al., 2015; Schmidt et al., 2013), autophagy (Muñoz-Braceras et al., 2015) and phosphatidylinositol metabolism (Park et al., 2015); however, the molecular functions remain unclear. Mutations that cause ChAc usually result in a reduction or absence of VPS13A, but several cases of patients with amino acid substitutions have been described (reviewed in Rzepnikowska et al., 2017b). Mutations in the other *VPS13* genes are also associated with various neurological, mental and developmental disorders and intellectual disabilities (Fromer et al., 2014; Kolehmainen et al., 2003; Lesage et al., 2016). Studies have also reported links between mutations in the *VPS13* genes with diabetes (Grarup et al., 2011; Saxena et al., 2010) and with cancer (Furukawa et al., 2011; Morisaki et al., 2014). Currently, there is not an effective therapy for neurodegenerative disorders linked to *VPS13* mutations.

There is a single Vps13 protein of 3144 amino acid residues (aa) in the yeast *Saccharomyces cerevisiae.* This yeast Vps13 protein shares the highest degree of similarity (in terms of domain structure) with human VPS13A. The yeast *VPS13* gene was initially identified in a screen for mutants that secrete the vacuolar enzyme carboxypeptidase Y (CPY; EC 3.4.16.1), which implies a role in protein targeting to the vacuole (Bankaitis et al., 1986). Further studies, including those modelling mutations identified in patients, showed the importance of Vps13 in vesicular transport, particularly for Golgi-to-vacuole transport (Brickner and Fuller, 1997; De et al., 2017; Redding et al., 1996; Rzepnikowska et al., 2017a), endosomal trafficking (Dalton et al., 2017; Luo and Chang, 1997; Rzepnikowska et al., 2017a) and mitochondrial DNA maintenance (Park et al., 2016). Furthermore, it was recently shown that Vps13 is present at membrane contact sites – zones of physical contact between two organelles or between an organelle and a plasma membrane, which mediate direct transport of lipids, ions and metabolites. So far, Vps13 has been identified at the nuclear-vacuolar junction (NVJ), at the endosomal-mitochondrial junction (EMJ) and at the vacuolar-mitochondrial junction (v-CLAMP) (Lang et al., 2015; Park et al., 2016; reviewed in Rzepnikowska et al., 2017b). Vps13 is able to bind to phosphatidylinositol lipids via four different sites: N-terminal; C-terminal; and internal SHR-BD and APT1 domains (De et al., 2017; Rzepnikowska et al., 2017a). In addition, the null mutant exhibits a severe sporulation defect (Brickner and Fuller, 1997) due to involvement of Vps13 in formation of the prospore membrane (Nakanishi et al., 2007; Park and Neiman, 2012). Finally, Vps13 interacts with actin and actin cytoskeleton proteins, and has an impact on the actin cytoskeleton organisation (Michelot et al., 2010; Rzepnikowska et al., 2017a). Since actin patches are sites of endocytosis, defects in the functioning of the actin cytoskeleton are accompanied by a defect in endocytosis in *vps13*Δ cells (Luo and Chang, 1997; Rzepnikowska et al., 2017a), as observed in several other actin cytoskeleton mutants (Huckaba et al., 2004).

Endocytosis is a process that enables the uptake of extracellular materials and is involved in the regulation of plasma membrane composition. After initiation of endocytosis, an invagination of the plasma membrane is formed, followed by scission and formation of an endocytic vesicle. The force needed for membrane invagination is mainly produced during assembly of actin filaments at the endocytic site. One of the key players during this step is the Arp2/3 complex (reviewed in Goode et al., 2015). This complex is an actin nucleation factor and gives rise to branched actin filaments, facilitating a burst of actin assembly. The action of Arp2/3 is supported by a group of proteins called nucleation-promoting factors; among them are Las17, type I myosins and Abp1 (reviewed in Goode et al., 2015). In *S.* *cerevisiae* there are two type I myosins encoded by the homologous *MYO3* and *MYO5* genes. A single deletion of either *MYO3* or *MYO5* results in minor defects; however, the double-knockout mutant shows severe defects in actin polymerisation that result in impaired endocytosis and growth (Geli and Riezman, 1996; Goodson et al., 1996). In Myo3/5, several regions can be identified: a motor head domain; a linker region; and a tail, which promotes actin nucleation (Anderson et al., 1998). Recruitment of Myo5 to endocytic sites is regulated by calmodulin, a highly conserved, calcium-binding, regulatory protein of 147 aa (Grötsch et al., 2010). Calmodulin binds to IQ motifs found in the linker region of Myo5 (Geli et al., 1998). Upon binding of calmodulin, Myo5 changes its conformation from open to closed, in which form it is unable to bind to membranes and perform its functions (Geli et al., 1998). At endocytic sites, Myo5 facilitates membrane internalisation via both motor and nucleation functions (Sun et al., 2006). Calmodulin also binds to the Arc35 subunit of the Arp2/3 complex (Schaerer-Brodbeck and Riezman, 2003), and to the Rvs167 endocytic protein involved in vesicle scission (Myers et al., 2016). Thus, endocytosis and the actin cytoskeleton organisation are regulated by calmodulin in several ways.

Calcium ions (Ca^2+^) are one of the most crucial signalling molecules that enable cells to adapt to changes in internal and external conditions. Cytosolic Ca^2+^ concentration is maintained at low levels, 50-200 nM (Iida et al., 1990; Nakajima-Shimada et al., 1991), and is regulated by a system of calcium pumps present in the membranes. In yeast, Ca^2+^ is stored mainly in vacuoles (Dunn et al., 1994), but also in the Golgi apparatus and endoplasmic reticulum (ER) (Pezzati et al., 1997). Changes in cytosolic Ca^2+^ concentration influences the state of calmodulin, which can be present in Ca^2+^-free or Ca^2+^-bound forms. The different forms have different protein targets (reviewed in Cyert, 2001; Yap et al., 1999). One of the Ca^2+^-independent targets is Myo5. Among Ca^2+^-dependent targets, there is a protein phosphatase calcineurin (reviewed in Cyert, 2001) and Arc35 (Schaerer-Brodbeck and Riezman, 2003). Calcineurin is a heterodimer consisting of catalytic and calmodulin-binding subunit A – which, in *S. cerevisiae*, is encoded by two redundant genes, *CNA1* and *CMP2* (*CNA2*) – and a regulatory and calcium-binding subunit B, encoded by the *CNB1* gene (Cyert et al., 1991; Cyert and Thorner, 1992; Liu et al., 1991). Upon activation by calmodulin, calcineurin mediates the response to changes in Ca^2+^ concentration through its main target, Crz1 (Matheos et al., 1997; Stathopoulos and Cyert, 1997). Crz1 is a transcription factor, which, after dephosphorylation by calcineurin, translocates to the nucleus and binds to calcineurin-dependent response elements (CDREs), enabling calcineurin-driven gene expression (Stathopoulos-Gerontides et al., 1999; Stathopoulos and Cyert, 1997). Calmodulin and calcineurin are highly conserved in eukaryotes, are crucial for calcium signalling (reviewed in Cyert, 2001), and are essential for the function and development of the central nervous system. Expression of calcineurin catalytic subunits in the brain is significantly higher than in other organs (Bond et al., 2017) and mutations in these subunits or changes in activity, both down- and upregulation, result in neurological disorders (reviewed in Kipanyula et al., 2016; Mizuguchi et al., 2018). It was also reported recently that calcium signalling is defective in cells isolated from ChAc patients (Pelzl et al., 2017a,b). Thus, components of the calcium signalling pathway are potential targets in therapy for neurodegenerative diseases.

Studies of yeast *vps13* mutant, a model for ChAc and other diseases connected with *VPS13* genes, are ongoing. Recently, missense mutations identified in ChAc patients were modelled in yeast, giving insight into possible mechanisms of pathogenesis (Park et al., 2016; Rzepnikowska et al., 2017a). Here, we describe a new growth phenotype of *vps13* mutants, which we found suitable for genetic screens and drug testing. Our genomic library screen revealed a *MYO3* gene fragment as a multicopy suppressor of *vps13-I2749R* and *vps13*Δ phenotypes, proving that both point mutations in, and deletions of, *VPS13* could be overcome. Further genetic and biochemical analyses pointed to calcium signalling, with the involvement of calmodulin and calcineurin, as an important factor for the alleviation of *vps13* defects. Our new phenotype could be useful for drug screening, as demonstrated by testing with calcineurin inhibitors. These findings can help to better understand the mechanisms of ChAc and will help to develop new treatments in the future.

## RESULTS

### Inactivation of *VPS13* causes hypersensitivity to sodium dodecyl sulphate in yeast cells

**Fig. 1.**
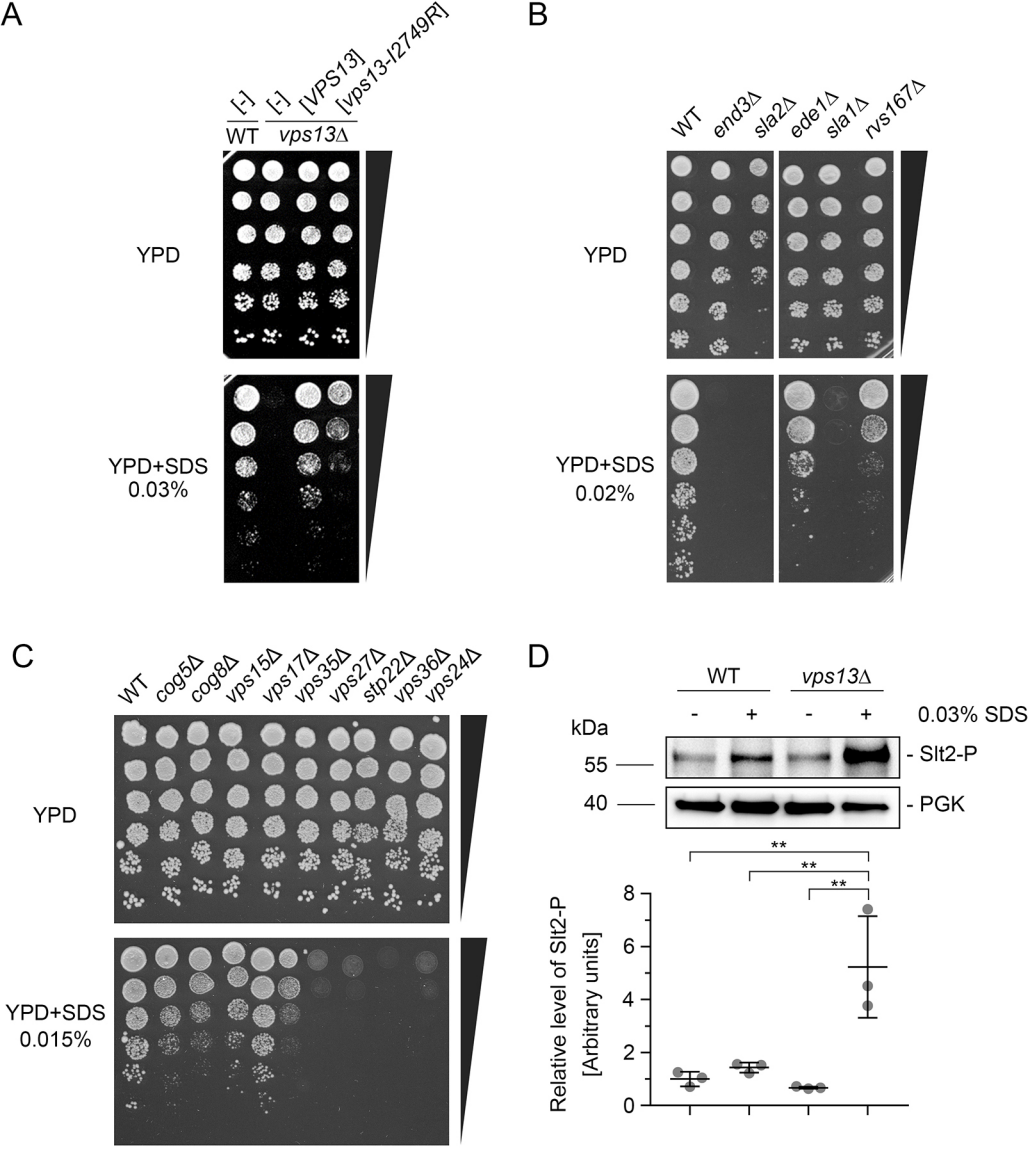
**The *vps13* mutants are hypersensitive to SDS but cell wall integrity is not compromised.** (A) *vps13* mutants are hypersensitive to SDS. (B) Mutants defective in endocytosis are hypersensitive to SDS. (C) Mutants defective in Golgi transport or endocytic sorting are hypersensitive to SDS. (D) Slt2 is hyperphosphorylated in *vps13* cells following SDS stress. Overnight yeast cultures were shifted to fresh YPD or YPD+0.03% SDS and incubated for 3 h. Western blot was performed and analysed by densitometry. The phosphorylation levels of Slt2 in relation to Pgk1 were compared by one-way ANOVA followed by Tukey's multiple comparison test (*n*=3); **P<0.01. Error bars indicate s.d.

In our previous study we discovered that *vps13* mutant cells, either *vps13*Δ or *vps13-I2749R*, with a single amino acid substitution in the APT1 domain of Vps13 corresponding to the I2771R mutation found in a ChAc patient, exhibit defects in actin cytoskeletal organisation and endocytosis (Rzepnikowska et al., 2017a). Various mutants that exhibit defects in the actin cytoskeleton, in protein transport, in cell wall integrity or have altered calcium homeostasis are hypersensitive to low concentrations of sodium dodecyl sulphate (SDS) (Ammons et al., 2015; Chen et al., 2012; Fokina et al., 2012; Varelas et al., 2006), a small amphiphilic detergent. Therefore, we tested both *vps13*Δ and *vps13-I2749R* mutants for growth in the presence of this compound. Both *vps13* mutants appeared hypersensitive to the addition of 0.03% SDS to the rich medium (Fig. 1A), and *vps13*Δ was most sensitive. In addition, we found that other mutants that are defective in actin cytoskeleton organisation and endocytosis (*end3*Δ, *sla2*Δ, *ede1*Δ, *sla1*Δ and *rvs167*Δ), or defective in Golgi transport or endocytic sorting (*cog5*Δ, *cog8*Δ, *vps15*Δ, *vps35*Δ, *vps27*Δ, *stp22*Δ, *vps36*Δ and *vps24*Δ), are also hypersensitive to low concentrations of SDS (Fig. 1B,C). Under SDS stress conditions, Golgi and endocytic traffic are probably crucial for the effective replacement of plasma membrane proteins that have been damaged by SDS.

It was previously documented that SDS damages the cell wall and plasma membrane in *Pichia pastoris* yeast cells (reviewed in Levin, 2005; Zhang et al., 2015); therefore, we investigated whether the cell wall integrity is compromised in *vps13*Δ. Cellular integrity in yeast is mainly controlled by the cell wall integrity (CWI) pathway (reviewed in Levin, 2005), which employs one branch of a mitogen-activated protein kinase (MAPK) cascade for signal transduction. This MAPK signalling pathway involves protein kinase C, Bck1, Mkk1/2 and Slt2/Mpk1 kinase, which further phosphorylates and activates transcription factors to produce proteins involved in cell wall biogenesis and other processes. Increased phosphorylation of Slt2 usually indicates a defect in cell wall integrity. Investigation of the level of phosphorylated Slt2 by western blotting using anti-phospho-p44/42 MAPK antibody in *vps13*Δ cell extracts revealed that levels were similar to the wild type. This finding supports the view that the CWI pathway is not activated and cell wall integrity is not compromised in *vps13*Δ cells grown in regular growth medium (Fig. 1D). Addition of SDS to the medium activated the CWI pathway in *vps13*Δ cells with approximately 5-fold higher phosphorylation of Slt2 kinase compared to wild type (Fig. 1D). This activation is apparently not sufficient to compensate for the damage, caused by SDS, to *vps13*Δ mutant cells.

### A fragment of the *MYO3* gene suppresses the SDS hypersensitivity of *vps13-I2749R* and *vps13*Δ cells

**Fig. 2.**
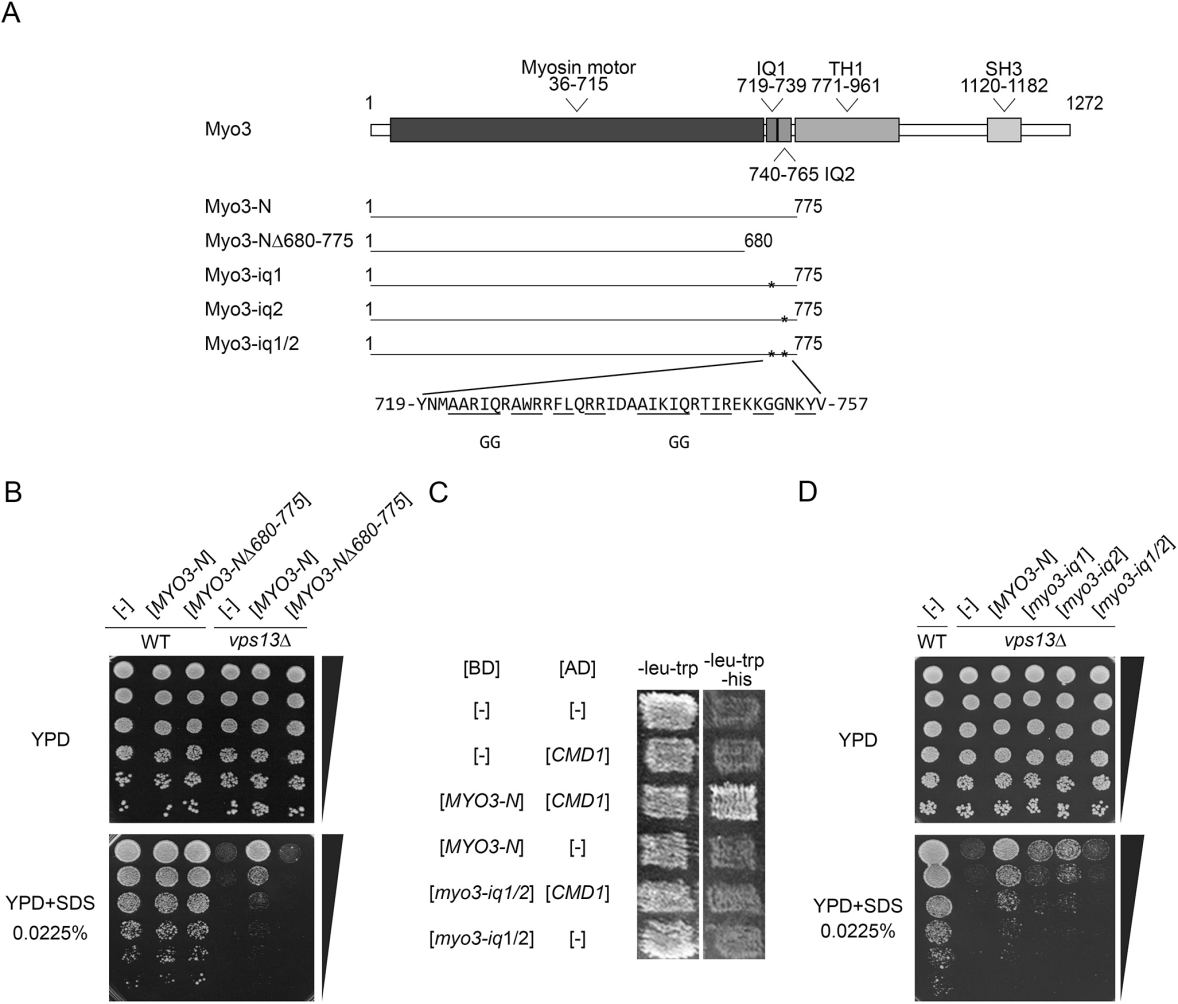
**The *MYO3-N* fragment encoding the motor domain and calmodulin-binding motifs from Myo3 suppresses hypersensitivity of *vps13*Δ to SDS.** (A) Schematic representation of Myo3 domain structure and Myo3 variants studied. Myo3 domains and motifs are based on the Uniprot database (http://www.uniprot.org/uniprot/P36006, 02.08.2018). *, position of mutations introduced *in vitro*. Amino acids conforming to the consensus of the calmodulin-binding motif as defined in the SMART database (http://smart.embl-heidelberg.de, 02.08.2018). IQ motifs are underlined and amino acid substitutions are shown. (B) *MYO3-N* suppresses *vps13*Δ but *MYO3-N*Δ*680-775* does not. (C) Myo3-N interacts with calmodulin in the two-hybrid system and mutations in IQ motifs abolish this interaction. BD, DNA-binding domian; AD, activating domain. (D) IQ motifs of Myo3 are required for suppression of *vps13*Δ.

To determine whether *vps13* mutations can be overcome, and to elucidate the importance of Vps13-dependent processes in SDS tolerance, we performed a screen for multicopy suppressors of *vps13-I2749R*. The *vps13*Δ strain carrying the *vps13-I2749R* allele on a plasmid was transformed with a multicopy genomic library. Several clones, bearing various none-overlapping genomic fragments, were able to grow on SDS-containing medium, and plasmids from these clones were isolated, retested for suppression and sequenced. One plasmid contained a fragment of the *MYO3* gene (*MYO3-N*) encoding the N-terminal 1-775 aa of type I myosin (Myo3-N), an actin cytoskeleton protein involved in endocytosis (Geli and Riezman, 1996; Goodson and Spudich, 1995), which was responsible for suppression. The Myo3-N fragment consists of: a motor domain (aa 36-715); a linker (aa 719-771), containing two IQ motifs, indicating ability to bind calmodulin (Ho et al., 2002); and five amino acids of a tail lipid-binding TH1 domain (aa 771-775) (Fig. 2A). Subsequently, we found that the *MYO3-N* fragment also suppresses the *vps13*Δ deletion mutation (Fig. 2B). This suggests that the Myo3-N protein does not suppress defects by directly interacting with Vps13-I2479R, but instead may involve another factor. Furthermore, we showed that a fragment of Myo3-N containing the IQ motifs (aa 680-775) was necessary for suppression (Fig. 2B), suggesting that calmodulin could be this other factor.

To verify the hypothesis that calmodulin binding by Myo3-N is required for suppression, three *myo3-N* alleles with one (*myo3-iq1*, *myo3-iq2*) or both (*myo3-iq1/2*) IQ motifs disrupted were generated by *in vitro* mutagenesis (Fig. 2A). To test the interaction of Myo3-N and Myo3-iq1/2 with calmodulin, we used a two-hybrid system, which was previously shown to be appropriate for the detection of the interaction between Myo5 (a homologue of Myo3) and calmodulin (Geli et al., 1998). As predicted, Myo3-N interacted with calmodulin but Myo3-iq1/2 protein did not (Fig. 2C). Most importantly, the *myo3-iq1/2* allele was not able to suppress the SDS-hypersensitivity phenotype of *vps13*Δ (Fig. 2D), although it was similarly expressed as *MYO3-N*, when the cellular levels of hemagglutinin (HA)-tagged versions of protein products were compared (Fig. S1). The *myo3-iq1* and *myo3-iq2* mutant alleles, each with a single IQ motif removed, were less efficient in suppression than *MYO3-N* (Fig. 2D). The additive effects of IQ deletions were previously observed for *MYO5* (Geli et al., 1998). Our results indicate that the mechanism by which *MYO3-N* suppresses *vps13*Δ requires binding of Myo3-N to calmodulin and that calmodulin signalling affects the *vps13*Δ phenotype.

### Crz1-dependent transcriptional response is increased in *vps13*Δ

**Fig. 3.**
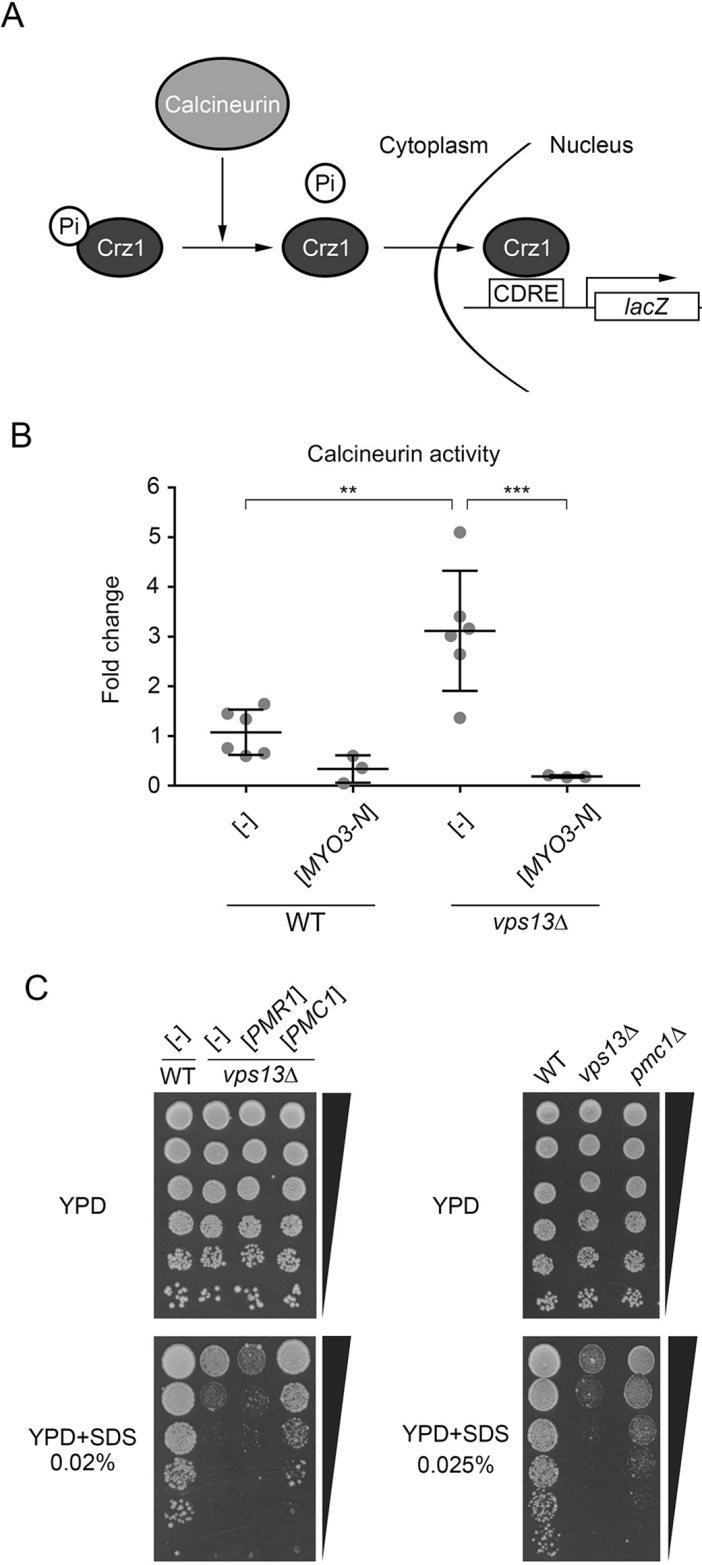
**Activity of calcineurin is increased in *vps13*Δ and reduced by *MYO3-N*, and *PMC1* suppresses the *vps13*Δ growth defect.** (A) Schematic representation of the calcineurin-Crz1 signalling system used to measure the activity of calcineurin. (B) Activity of calcineurin is increased in *vps13*Δ and is diminished by *MYO3-N*. Results were analysed statistically by one-way ANOVA followed by Tukey's multiple-comparisons test (*n*=6 for WT [-] and *vps13* [-], *n*=3 for WT [*MYO3-N*] and *vps13*Δ [*MYO3-N*]; **P<0.01, ***P<0.001). Error bars indicate s.d. (C) *PMC1* is a multicopy suppressor of *vps13*Δ hypersensitivity to SDS and *pmc1*Δ is hypersensitive to SDS. Growth of wild-type and *vps13*Δ transformed with plasmids bearing *PMR1* or *PMC1*, or with empty vector (left panel). Growth of wild-type, *vps13*Δ and *pmc1*Δ strains on SDS medium (right panel).

Calmodulin has several targets in yeast, including the protein phosphatase calcineurin, which is important in the response of cells to stress (reviewed in Cyert, 2001). In response to increased cytoplasmic calcium upon stress, calmodulin binds and activates calcineurin, which subsequently dephosphorylates and activates the transcription factor Crz1 (Matheos et al., 1997; Stathopoulos and Cyert, 1997) to promote cell survival. To compare activity of calcineurin in wild-type and *vps13*Δ cells, and in these cells upon expression of *MYO3-N*, we used a plasmid containing a calcineurin-dependent response elements (CDREs) fused to the *lacZ* open reading frame encoding β-galactosidase (Stathopoulos and Cyert, 1997) (Fig. 3A). Activity of β-galactosidase was measured in cell extracts derived from respective transformants. Results show that the activity of calcineurin is significantly increased in *vps13*Δ when compared to the wild-type strain (Fig. 3B) and it is reduced by the expression of *MYO3-N* (Fig. 3B). These results imply that cytoplasmic calcium concentration could be increased in *vps13*Δ and that Myo3-N might act by binding and sequestering calmodulin, which in turn causes deactivation of calcineurin. This suggests that deactivation of calcineurin by genetic manipulation or by chemical inhibitors might possibly cause suppression.

### *PMC1* gene, encoding vacuolar calcium pump, is a multicopy suppressor of *vps13*Δ

To address the question of whether an increased cytoplasmic calcium level is the reason for hyperactivation of calcineurin in *vps13*Δ, we tested the effect of overexpression of *PMR1* and *PMC1* genes for growth of *vps13*Δ on SDS medium. These genes encode ER/Golgi or vacuolar calcium pumps, respectively, and transport calcium out of the cytoplasm. We found that *PMC1*, but not *PMR1*, is a potent suppressor (Fig. 3C). We analysed further whether the defect of vacuolar transport of calcium in *pmc1*Δ is alone sufficient to decrease yeast cell tolerance to SDS and found that *pmc1*Δ is hypersensitive to SDS but less than *vps13*Δ (Fig. 3C). Since *vps13*Δ shows defects in Golgi-to-vacuole transport, the pathway needed for normal Pmc1 targeting, the Pmc1 pump could possibly be mislocalised in these cells. Thus, mislocalisation of Pmc1 could be one of the reasons for *vps13*Δ hypersensitivity to SDS. However, we were unable to prove this since we could not get *PMC1-mCherry vps13*Δ clones. This suggests that such cells might be unviable.

### The fine-tuned activity of calcineurin is important for *vps13*Δ growth on SDS-containing medium

**Fig. 4.**
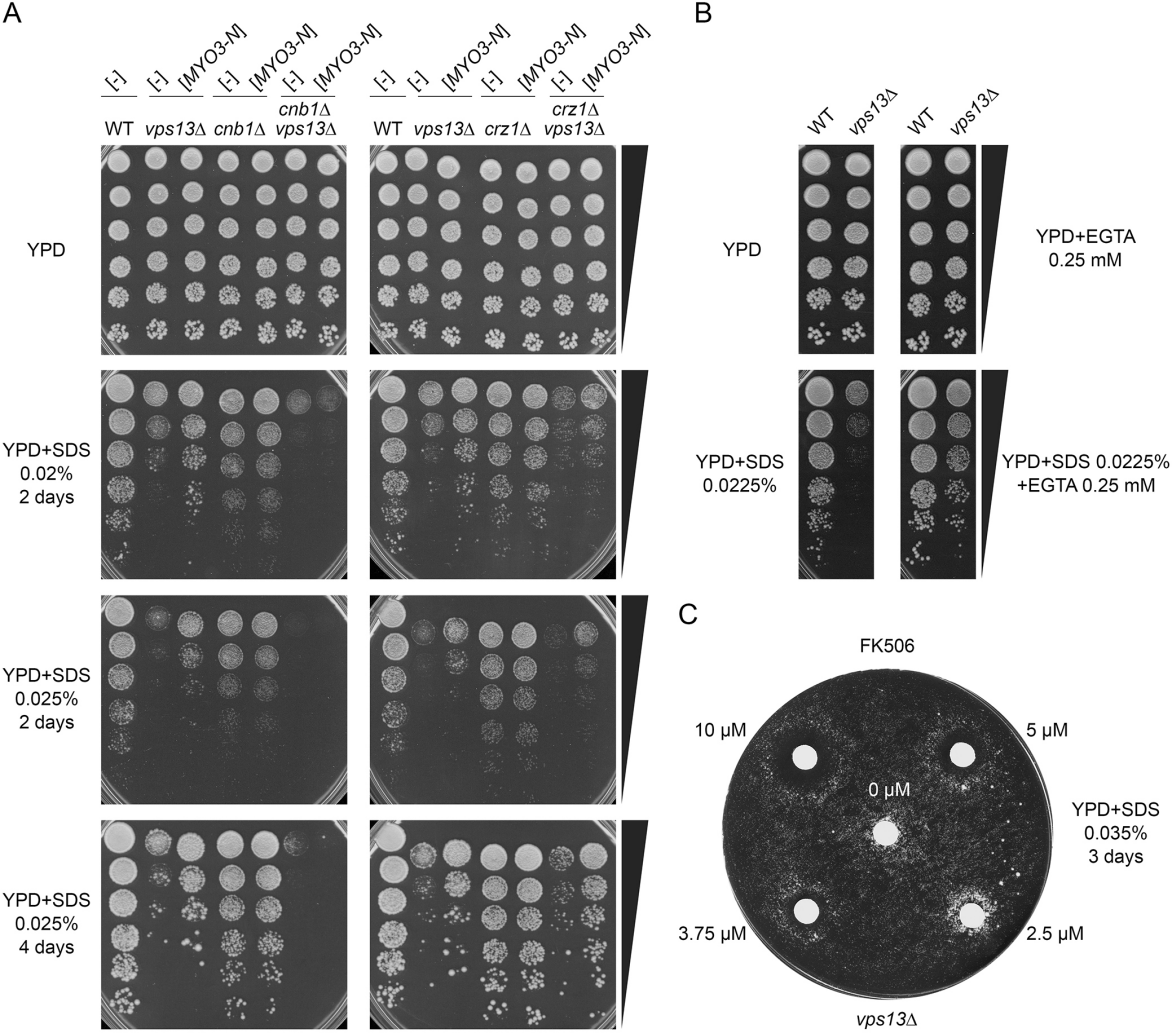
**A normal/intermediate activity of calmodulin-calcineurin pathway is required to cope with SDS stress in *vps13*Δ.** (A) Growth of wild-type, *vps13*Δ, *cnb1*Δ, *crz1*Δ, *vps13*Δ *cnb1*Δ and *vps13*Δ *crz1*Δ cells expressing, or not, *MYO3-N* on YPD+SDS. (B) Addition of EGTA improves growth of *vps13*Δ on SDS-containing medium. (C) FK506 affects growth of *vps13*Δ on SDS-containing medium. Strains were grown to early exponential phase. Cells (0.05 OD) were plated on YPD+0.035% SDS, and 2 µl of 0-10 µM FK506 (HY-13756, MedChemExpress, Monmouth Junction, USA) was applied on paper discs as indicated.

The Cnb1 regulatory subunit of calcineurin is required for its activity (Cyert and Thorner, 1992). It has been reported that the *cnb1*Δ strain is more sensitive to a variety of stresses (Mulet et al., 2006), but there is no data relating to the sensitivity to SDS. Therefore, to analyse how calcineurin deactivation affects the response to SDS in *vps13*Δ cells and to establish whether active calcineurin is required for *vps13*Δ suppression by *MYO3-N*, the growth of wild-type, *cnb1*Δ and *cnb1*Δ *vps13*Δ mutants on SDS-containing medium was compared in the presence or absence of *MYO3-N.* We found that the *cnb1*Δ strain is more sensitive to SDS than the wild-type strain and that *cnb1*Δ mutation negatively interacts genetically with *vps13*Δ (Fig. 4A), indicating that *vps13*Δ cells rely on active calcineurin to cope with SDS stress. Interestingly, the *cnb1*Δ *vps13*Δ mutant was not suppressed by *MYO3-N*, indicating that Myo3-N requires the Cnb1 protein and functional calcineurin for *vps13*Δ suppression (Fig. 4A). Thus, it seems that a certain level of calcineurin activity is required for normal growth in the presence of SDS. Sequestration of calmodulin, the activator of calcineurin, by *MYO3-N* results in a reduction in calcineurin activity, thus fine tuning calcineurin activity to this required level.

The contribution of Crz1 to the SDS stress response and to the *MYO3-N* suppression of *vps13*Δ was also studied. Comparison of the growth of isogenic *crz1*Δ and *crz1*Δ *vps13*Δ mutants with *vps13*Δ and with the wild-type strain revealed that the *crz1*Δ strain exhibits a similar sensitivity to SDS as the wild-type strain in conditions tested. It also revealed that *crz1*Δ *vps13*Δ grows more slowly than *vps13*Δ but suppression by *MYO3-N* is still observed (Fig. 4A). These results show that the Crz1 transcription factor positively contributes to SDS stress tolerance in *vps13*Δ and, most importantly, that *vps13*Δ suppression by *MYO3-N* involves a mechanism that is independent of Crz1 activity.

To investigate the possibility of using chemical intervention in the *vps13*Δ strain, we tested the effects of: ethylene glycol tetraacetic acid (EGTA), a chelator of divalent cations that is more specific for Ca^2+^ than for other cations; and FK506, a calcineurin inhibitor. EGTA reduces the concentration of calcium in the cytoplasm, thus inhibiting the Ca^2+^-dependent action of calmodulin and inhibiting calcineurin. Interestingly, addition of EGTA significantly improved growth of *vps13*Δ on SDS plates (Fig. 4B). Thus, a partial inhibition of the calcium-dependent functions of calmodulin and the inhibition of calcineurin is beneficial for *vps13*Δ cells in the presence of SDS stress. FK506 improved growth of *vps13*Δ at low concentrations (2.5 μM spotted onto filter paper), but higher concentrations exhibited an adverse effect (Fig. 4C). Thus, calcineurin cannot be inhibited completely, but its downregulation can be fine-tuned in order to improve the growth of *vps13* cells. This result points to FK506 as a potential drug and to calcineurin as a useful target to select drugs for ChAc therapy.

### The *cmd1-226* mutant allele, encoding a calmodulin variant with the F92A amino acid substitution, suppresses *vps13*Δ

**Fig. 5.**
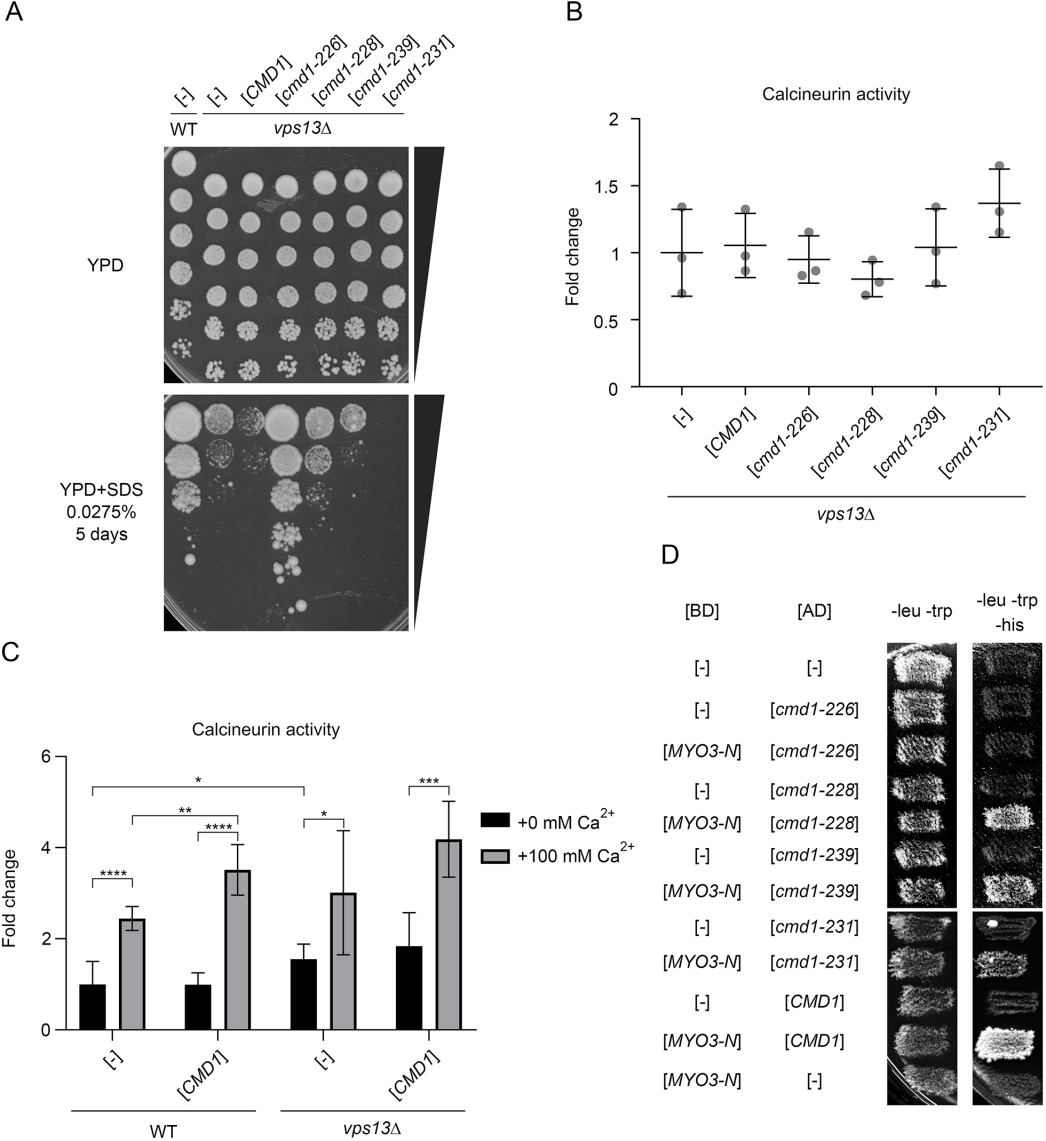
**The *cmd1-226* mutant allele, but not wild-type *CMD1*, restores growth of *vps13*Δ on YPD+SDS plates.** (A) Different *CMD1* alleles variously affect the growth of *vps13*Δ. (B) Wild-type and mutant calmodulin do not influence calcineurin activity in *vps13*Δ cells. Results were analysed by one-way ANOVA (*n*=3); *P*=0.196. Error bars indicate s.d. (C) Effects of calcium addition and *CMD1* overexpression on calcineurin activity in wild-type and *vps13*Δ cells. Results were analysed statistically by two-tailed Student's *t*-test (*n*=6; *P<0.05, **P<0.01, ***P<0.001, ****P<0.0001. Error bars indicate s.d. (D) Cmd1-226 does not interact with Myo3-N. Results from a two-hybrid experiment are shown. BD, DNA-binding domain; AD, activating domain.

The importance of calmodulin binding by Myo3-N for the suppression of *vps13* suggests that a calmodulin sequestration mechanism is involved. To verify this assumption, we analysed whether expression of multiple copies of *CMD1* alleles encoding wild-type or mutant calmodulin have an effect on the growth of *vps13*Δ cells in the presence of SDS. The various *cmd1* alleles were selected to represent different groups of mutants with the same phenotype: defective actin cytoskeleton, altered localisation of calmodulin, defective karyokinetic spindle or defective budding, as previously described (Geli et al., 1998; Ohya and Botstein, 1994). The wild-type *CMD1* negatively affected growth of *vps13*Δ cells, implying that the control of the calmodulin pool and/or accessibility to different substrates is important for regulation of the cell response to SDS stress in *vps13*Δ, supporting the sequestration mechanism for *MYO3-N* (Fig. 5A). The *cmd1-231* allele, which causes a defect in budding, affected growth similarly to wild-type *CMD1*, while the mutant *cmd1-228* and *cmd1-239* alleles essentially did not affect growth of *vps13*Δ. Surprisingly, the *cmd1-226* allele, which results in the F92A substitution in calmodulin and represents a group of mutants that show defects in the organisation of the actin cytoskeleton and in endocytosis, was a potent suppressor of *vps13*Δ (Fig. 5A).

To test how overproduction of wild-type or mutant calmodulin affects activity of calcineurin in *vps13*Δ cells, the *CDRE-lacZ* reporter was used*.* Interestingly, neither wild-type nor mutant calmodulin genes, including the *cmd1-226* suppressor allele, significantly affected the increased activity of calcineurin in *vps13*Δ cells (Fig. 5B). Since additional calmodulin did not have an effect in *vps13*Δ, we checked whether calcineurin could be stimulated by addition of calcium to the medium in this strain. Wild-type and *vps13*Δ cells showed higher activity of calcineurin after calcium addition, indicating that calcineurin activity in *vps13*Δ in regular conditions is not maximal (Fig. 5C). Moreover, in wild-type cells, the effect of calcium and expression of additional copies of *CMD1* was additive. However, *vps13*Δ cells expressing *CMD1* from the plasmid did not significantly increase calcineurin activity in the presence or absence of additional calcium. Thus, even when calcium is not a limiting factor, further activation of calcineurin by Cmd1 requires Vps13. These results indicate that reduction of calcineurin activity is probably not a mechanism for restoring growth of *vps13* cells on SDS plates mediated by *cmd1-226.* The Cmd1-226 mutant protein might target proteins other than calcineurin. One possibility is that Cmd1-226 is different in terms of its binding with Myo3, as is the case for Myo2 (Sekiya-Kawasaki et al., 1998). Therefore, we analysed how selected *cmd1* mutations affect the interaction of calmodulin with Myo3 using a two-hybrid system. This analysis shows that the *cmd1-226* mutation, in contrast to other mutations tested, results in total loss of interaction between calmodulin and Myo3-N (Fig. 5D). Thus, a complete loss of interaction between mutant calmodulin and Myo3 is required for *cmd1-226*-based suppression, the opposite to *MYO3-N*-based suppression of *vps13*Δ. Cmd1-226 may act by diluting wild-type calmodulin and activating full-length Myo3, or by other mechanisms.

### Expression of *MYO3-N* suppresses the actin cytoskeleton polarisation and endocytosis defects of *vps13*Δ

**Fig. 6.**
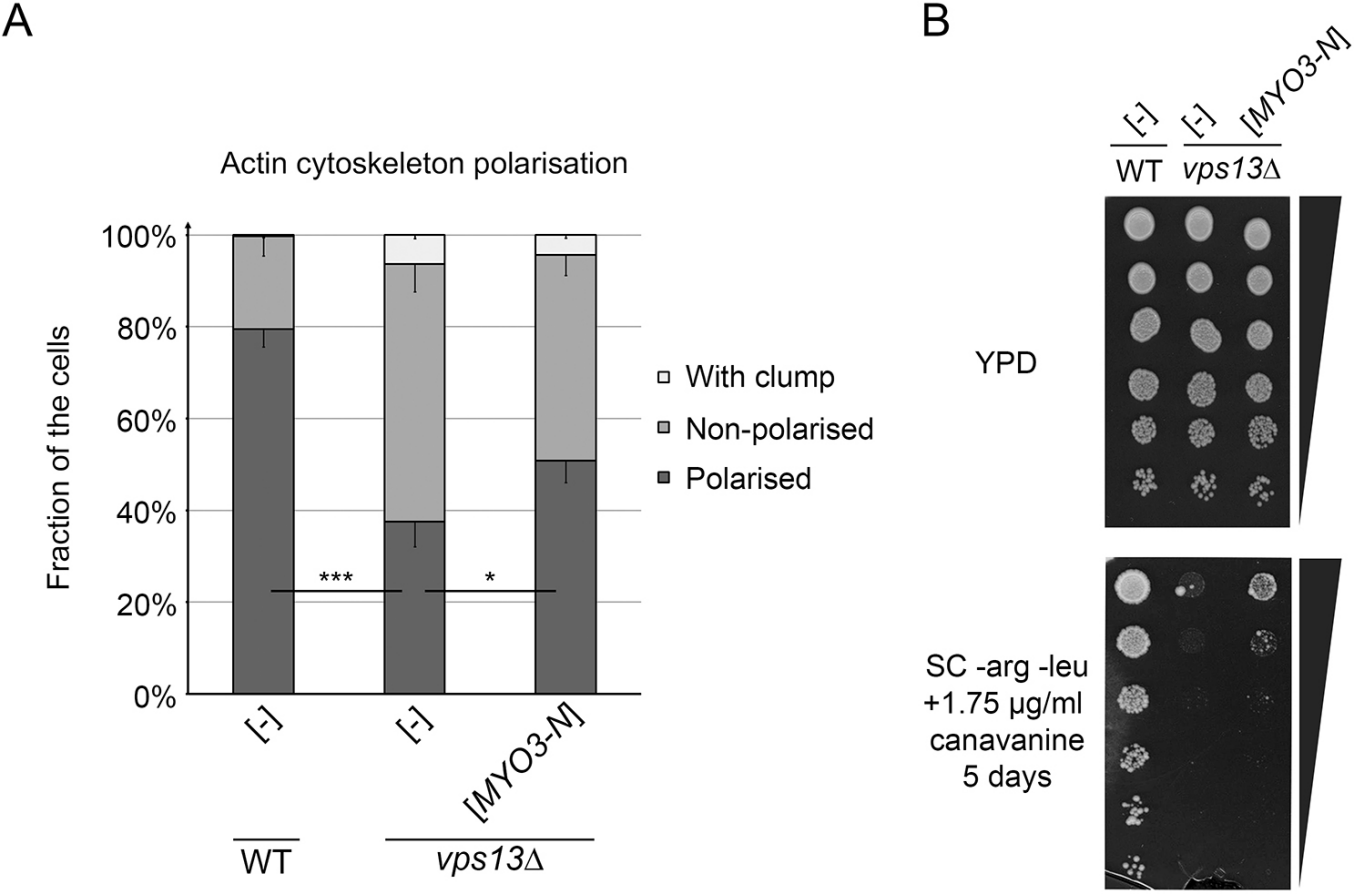
***MYO3-N* suppresses the actin-cytoskeleton polarisation defect and the canavanine hypersensitivity of *vps13* cells.** (A) *MYO3-N* suppresses the actin-cytoskeleton polarisation defect of *vps13*Δ. Quantification of actin polarisation was analysed using the two-tailed Student's *t*-test (*n*=3; cells counted per replicate: 105; 184; 148 for WT [-], 130; 103; 195 for *vps13*Δ [-], 138; 111; 121 for *vps13*Δ [*MYO3-N*]); *P<0.05, ***P<0.001. Error bars indicate s.d. (B) *MYO3-N* suppresses canavanine hypersensitivity of *vps13*Δ.

We have previously reported that *vps13*Δ shows defects in the organisation of the actin cytoskeleton that are accompanied by an endocytosis defect, observed as canavanine hypersensitivity (Rzepnikowska et al., 2017a). We found that *MYO3-N* suppresses the actin cytoskeleton polarisation defect of *vps13*Δ, since a greater number of cells with properly polarised actin patches in the bud, visible actin cables in the mother, and without actin clumps, were found when *MYO3-N* was expressed (Fig. 6A). The *vps13*Δ cells expressing *MYO3-N* were less sensitive to canavanine than *vps13*Δ cells bearing empty vector (Fig. 6B), also indicating that endocytosis is improved.

**Fig. 7.**
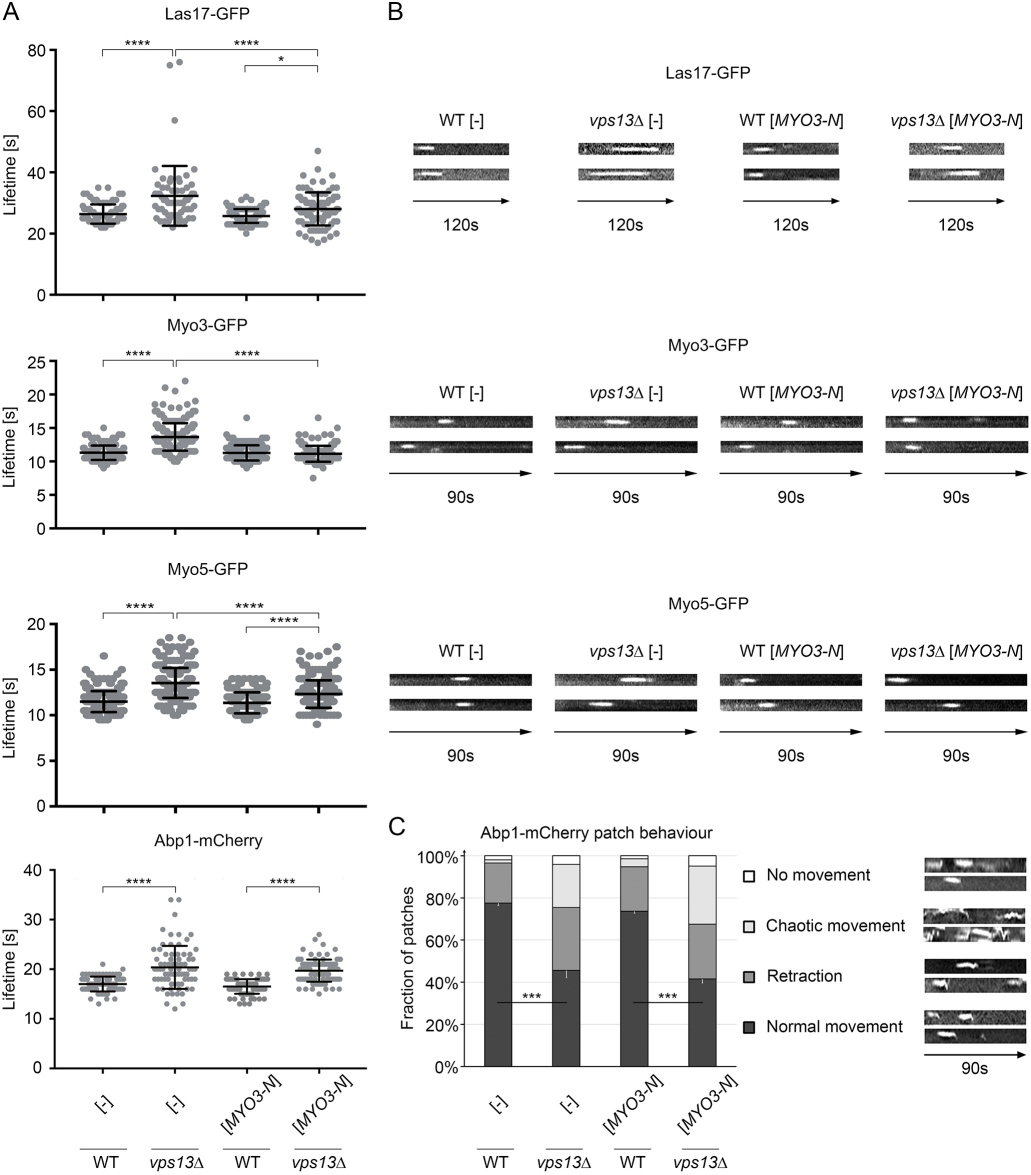
**The *vps13*Δ mutation impairs plasma membrane events involved in endocytosis and *MYO3-N* suppresses some of the defects.** (A) Lifetimes of Las17-GFP, Myo3-GFP, Myo5-GFP and Abp1-mCherry patches. Results were compared using one-way ANOVA followed by Tukey's multiple-comparisons test (*n*=87; 63; 103; 97 for Las17-GFP, *n*=163; 212; 207; 192 for Myo3-GFP, *n*=302; 264; 296; 251 for Myo5-GFP, *n*=83; 75; 104; 100 for Abp1-mCherry for WT [-], *vps13* [-], WT [*MYO3-N*] and *vps13* [*MYO3-N*], respectively); *P<0.05, ****P<0.0001. Error bars indicate s.d. (B) Kymographs of Las17-GFP, Myo3-GFP and Myo5-GFP patches. Two representative kymographs for each strain, as in A, are shown. Las17-GFP patches were observed for 120 s using an Olympus IX-81 microscope and imaging was performed via 1 s time lapse. Myo3-GFP and Myo5-GFP were observed for 90 s using an OMX DeltaVision V4 microscope and imaging was performed via 0.5 s time lapse. (C) Kymographs of Abp1-mCherry patches. Two representative kymographs for each class of patch behaviour are shown. Abp1-mCherry patches were viewed for 90 s using an Olympus IX-81 microscope and imaging was performed via 1 s time lapse. Quantification of the patches was analysed using a two-tailed Student's *t*-test (*n*=5, patches counted per replicate: 44; 45; 54; 55; 65 for WT [-], 50; 47; 60; 47; 60 for *vps13*Δ [-], 51; 57; 64; 37; 61 for WT [*MYO3-N*], 54; 69; 59; 67; 59 for *vps13*Δ [*MYO3-N*]); ***P<0.001. Statistical significances and error bars indicating s.d. are shown for patches with normal behaviour.

Actin patches are sites where endocytosis takes place (Huckaba et al., 2004). Vps13 has been found in artificial actin patches assembled on microbeads covered with the nucleation-promoting factor Las17 (yeast WASP) (Michelot et al., 2010). Therefore, it is possible that Vps13 is directly involved in the early steps of endocytosis and/or that the early steps of endocytosis in *vps13*Δ cells are disturbed by the actin cytoskeleton malfunctioning. To test this possibility, the lifetimes of endocytic reporters fused with a particular fluorescent protein (GFP or mCherry) in cortical patches were determined in the wild-type and *vps13*Δ strains. Kymographs, indicating the behaviour of individual patches, were also obtained. The reporter proteins analysed represent actin cytoskeleton components that associate temporarily with the endocytic vesicle as it forms. They are thus used for the visualisation of specific stages of endocytosis. Myo3/5 work together with Las17 in the myosin/WASP module to activate actin polymerisation (reviewed in Lu et al., 2016). Abp1 acts later in the actin module and is a marker that tracks invagination of the plasma membrane and inward endocytic vesicle movement. The actin cytoskeleton regulators Las17, Myo3, Myo5 and Abp1 were monitored by fluorescence microscopy. In *vps13*Δ cells, the lifetimes of the patches formed by all of these proteins were longer than in respective control cells, 32.32 for Las17-GFP, 13.66 for Myo3-GFP, 13.55 for Myo5-GFP and 20.37 s for Abp1-mCherry as opposed to 26.38, 11.29, 11.49 and 17.01 s, respectively (Fig. 7A). Lifetimes of Las17-GFP and Myo5-GFP were partially, but significantly, shortened when *MYO3-N* was expressed in *vps13*Δ (28.05 and 12.32 s, respectively) (Fig. 7A). *MYO3-N* did not affect the lifetime of Abp1-mCherry (19.7 s) (Fig. 7A). Interestingly, the lifetime of Myo3-GFP patches in *vps13*Δ cells expressing *MYO3-N* was shortened to a normal value (11.14 s) and was statistically not different from the control strain (Fig. 7A).

Kymographs suggested that the immobile phase of Las17-, Myo3-, Myo5- and Abp1-associated patches was affected *–* these proteins were retained for longer at the plasma membrane in *vps13*Δ than in the wild-type cells (Fig. 7B). What is more, the Apb1m-Cherry patch tracks indicate that the movement of some of the patches was affected in *vps13*Δ mutant cells (Fig. 7C). At least three abnormal behaviours of the patches were observed: the patch remained near the plasma membrane and did not move toward the centre of the cell (no movement); it moved inward but then retracted; or it moved in a direction other than towards the cell centre (chaotic movement). *MYO3-N* expression did not influence the defective patch movement of *vps13*Δ (Fig. 7C).

In summary, the results suggest that Vps13 is involved in the early steps of endocytosis and it affects the duration of association of several actin cytoskeleton proteins with endocytic sites. It also affects the movement of endocytic vesicles, as observed by Abp1-mCherry, in terms of occurrence, distance travelled and direction. Production of Myo3-N in *vps13*Δ improves endocytosis by correcting a number of steps in the process, in particular restoring the functioning of full-length Myo3.

### Osh2 and Osh3 proteins bridging cortical ER with plasma membrane at endocytic sites are essential for suppression of *vps13*Δ by *MYO3-N*

**Fig. 8.**
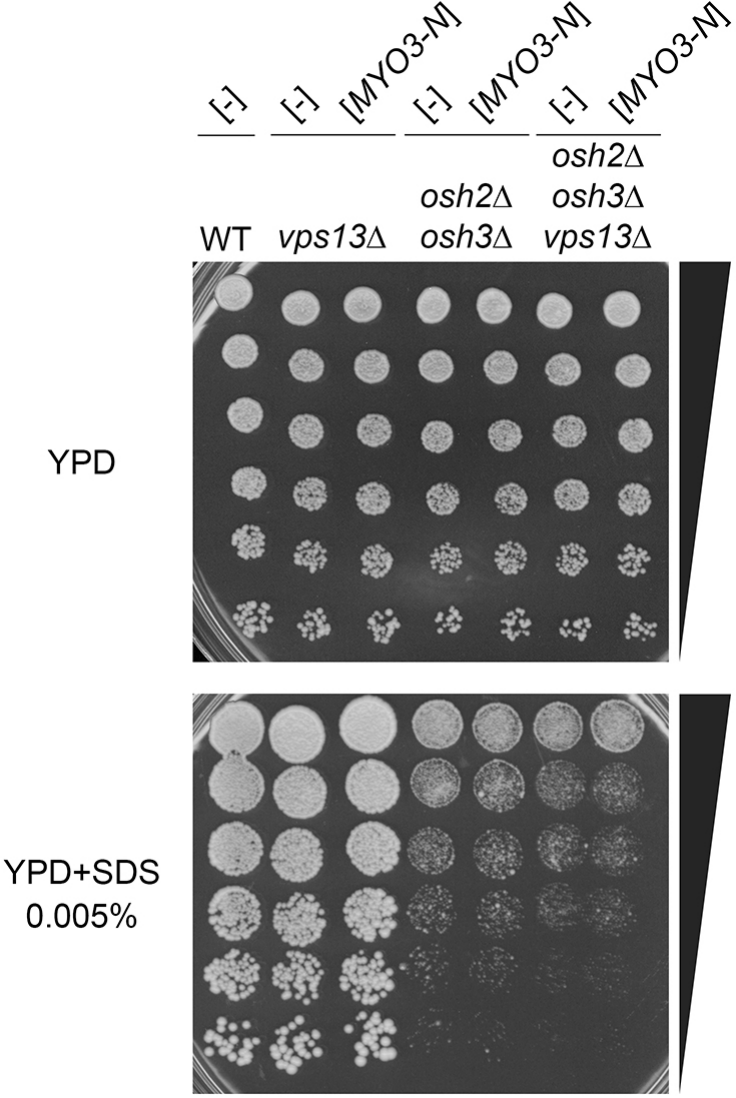
**The *osh2*Δ *osh3*Δ double mutant is hypersensitive to SDS and Osh2/3 are important for *vps13*Δ suppression by *MYO3-N*.**

We found that the defects of *vps13*Δ cells – in endocytosis, in the extended lifetime of Las17-GFP and in abnormal Abp1 patch behaviour – are similar to the defects observed in *osh2*Δ *osh3*Δ cells (Encinar del Dedo et al., 2017). Osh2 and Osh3 are components of ER-plasma membrane contact sites important for endocytosis (Encinar del Dedo et al., 2017). Therefore, we tested *osh2*Δ *osh3*Δ and other mutants defective in various membrane contact sites for growth in the presence of SDS and whether Osh2/3 proteins are required for *MYO3-N* suppression of *vps13*Δ. The *nvj1*Δ, *vps39*Δ and *mmm1*Δ mutants, lacking the proteins involved in the formation of nuclear-vacuolar junctions, v-CLAMPs and ER-mitochondria encounter structures (ERMES), respectively, grew as wild type on SDS-containing medium, showing that these membrane contact sites are not important in this condition (Fig. S2). The analysis of *osh2*Δ *osh3*Δ double mutants, and *osh2*Δ *osh3*Δ *vps13*Δ triple mutants, transformed with *MYO3-N* plasmid or empty vector, revealed that: the *osh2*Δ *osh3*Δ strain is much more sensitive to SDS than *vps13*Δ; the *osh2*Δ *osh3*Δ *vps13*Δ is similarly sensitive to *osh2*Δ *osh3*Δ; and neither is suppressed by *MYO3-N* (Fig. 8). Therefore, Osh2/3 are crucial for SDS tolerance and, under this stress condition, act with Vps13 in the same pathway. Finally, Osh2/3 are important for Myo3-N to act as a suppressor of *vps13*Δ. Thus, calmodulin-binding motifs of Myo3-N and the presence of full-length myosin type I linked with Osh2/3 at ER-plasma membrane endocytic contact sites, but not other membrane contact sites, are important for growth of the *vps13*Δ strain on SDS-containing medium.

## DISCUSSION

Vps13 proteins are important for neuronal cell physiology, as mutations in *VPS13* genes cause genetic neurodegenerative disorders in humans. Despite this importance, the molecular function of Vps13 and pathogenesis of associated diseases are still unclear. Using a yeast model for ChAc, a *vps13*Δ mutant and its newly discovered SDS-hypersensitivity phenotype, we have screened for genetic suppressors to see whether the Vps13 deficiency can be overcome and to learn more about the pathological cellular processes that could be the basis for the disease. Here, we show that *vps13*Δ defects can be suppressed by: *MYO3-N*, which encodes the Myo3-N myosin fragment that binds and sequesters calmodulin; expression of the *cmd1-226* allele encoding a mutant calmodulin that is unable to bind myosin; or by *PMC1*, encoding a vacuolar calcium pump. We also show that calcium signalling is activated in *vps13*Δ and that Myo3-N downregulates calcineurin activity and acts as a suppressor via a Crz1-independent mechanism. Furthermore, we show that *vps13*Δ can also be suppressed using chemical compounds, EGTA and FK506, which affect calcium signalling and inhibit calcineurin. Our studies of early endocytosis events in *vps13*Δ and genetic analysis point to the possibility that Vps13 is a component of ER–plasma-membrane contact sites.

Discovering a new phenotype of *vps13*Δ cells, hypersensitivity to SDS, allowed us to perform a successful screen for multicopy suppressors, but also has potential implications for ChAc patients. SDS is one of the most popular detergents worldwide. It is used as an additive to a wide range of cosmetics, pharmaceuticals, and household and industrial cleaning products. This amphiphilic compound easily accumulates in living organisms (reviewed in Cserháti et al., 2002), inhibits growth of bacteria, algae, fishes and yeasts (reviewed in Lechuga et al., 2016; Sandbacka et al., 2000), and negatively affects mammalian cells. The hypersensitivity of *vps13*Δ to SDS and the abundance of SDS in many everyday products suggest that ChAc patients may be more vulnerable to a danger posed by SDS. It could be one of the environmental factors that influence the time of onset and progression of the disease. However, sensitivity of human cells to SDS requires further study.

We used *vps13*Δ yeast as a ChAc model to better understand its pathophysiology. Our results show that the CWI signalling pathway was not activated in *vps13*Δ cells, suggesting that there is no defect in the cell wall in these cells. Rather, plasma membrane composition might be altered due to defects in endocytosis or plasma membrane stiffness/rigidity affected due to the defect in actin cytoskeleton organisation in *vps13*Δ. This may be the reason for low tolerance to SDS, as supported by the finding that several endocytic mutants with defects in the actin cytoskeleton and in endocytosis, and mutants defective in Golgi and endosomal sorting, were also found to be hypersensitive to SDS. We have found that the CWI pathway responds to SDS with a much greater level of phosphorylation in *vps13*Δ, when compared to wild-type cells, to compensate for damage caused by the detergent.

Changes in calcineurin activity are connected with several diseases, including neurodegeneration. Calcineurin signalling was elevated in *vps13*Δ, and this is detrimental, as shown for yeast models for other neurodegenerative diseases (Caraveo et al., 2014). One possible explanation is that a lack of Vps13 protein causes a defect in protein trafficking that triggers a cellular response involving calcineurin upregulation. Our data of *vps13*Δ suppression by *PMC1* suggest that Pmc1 could be mislocalised in *vps13*Δ and inefficient in pumping calcium to the vacuole, resulting in cytoplasmic calcium increase and calcineurin activation. Such a view is also supported by negative genetic interaction of *vps13*Δ with *pmr1*Δ (Costanzo et al., 2010). It is also possible that Vps13 is more directly involved in calcium signalling, since, in high-throughput studies, Vps13 has been shown to bind calmodulin (Ho et al., 2002). However, we were not able to confirm this interaction by other methods. Although detrimental in excess, the activities of calcineurin and of its Crz1 effector are important for the growth of *vps13*Δ cells under SDS stress, similarly to as observed for *Candida glabrata* yeast (Chen et al., 2012). Calcium signalling and calcineurin activity could be upregulated in *vps13* cells by at least two mechanisms: through secondary effects of disrupted transport processes or through the removal of potential direct interactions.

We have shown that the effects of the *vps13*Δ mutation, monitored through growth on SDS medium, can be overcome in several ways: titration of calmodulin by Myo3-N; expression of additional copies of *PMC1*; additional production of mutant calmodulin Cmd1-226; or addition of calcineurin inhibitors. Expressed Myo3-N binds to and sequesters free calmodulin, which activates Myo3/5 and downregulates the activity of calcineurin. The growth defect of the *cnb1*Δ *vps13*Δ mutant is not suppressed by *MYO3-N*; however, the *crz1*Δ *vps13*Δ mutant was still suppressed. This suggests that active calcineurin, but not Crz1, is required for the suppression and only partial downregulation is beneficial. This effect could be also achieved by lowering cytoplasmic calcium via overproduction of the Pmc1 vacuolar calcium pump. This notion is further supported by the finding that chemical calcineurin inhibitors, EGTA and FK506, suppressed the *vps13*Δ growth defect. FK506 suppressed *vps13*Δ only at a very low concentration, while at higher concentrations FK506 enhanced the *vps13*Δ hypersensitivity to SDS. Our results are in line with observations that the moderate calcineurin activity is protective for cells, but elevated or inhibited activity is toxic (Caraveo et al., 2014, reviewed in Kipanyula et al., 2016). In agreement with a calmodulin-sequestering model, overproduction of wild-type calmodulin is harmful to *vps13*Δ cells in spite of it not affecting calcineurin activity in *vps13*Δ cells. The *cmd1-226* also did not alter the calcineurin activity, which suggests that the mechanism of the suppression in this case is calcineurin independent. One possibility is that Cmd1-226 dilutes or somehow negatively affects wild-type calmodulin, what in turn increases the pool of the active forms of Myo3/5, which contributes to suppression by enhancing endocytosis. The other possible explanation of *cmd1-226* action is by interacting with Arc35, a subunit of the Arp2/3 complex that activates the polymerisation of actin filaments. Calmodulin encoded by *cmd1-226* binds to Arc35 protein, while that encoded by *cmd1-231*, which was the most toxic to *vps13*Δ, does not (Schaerer-Brodbeck and Riezman, 2003). We cannot exclude the possibility that SDS stress somehow causes cell division defects in *vps13*Δ and Cmd1-226 helps to overcome this defect by acting on other targets, such as Spc110, a spindle-pole-body calmodulin-binding protein (Geiser et al., 1993).

As we have documented here, *vps13*Δ exhibits defects in an early endocytic processes. These defects include an extended lifetime of several endocytic actin-cytoskeleton proteins and abnormal Abp1 patch behaviour, which is a marker of endocytic vesicle internalisation. Myo3-N production in *vps13*Δ results in restoration of the lifetime of Myo3 patches engaged in invagination and in release of endocytic vesicles, and in partially shortened lifetimes of Las17 and Myo5. Myo3-N production may also have other effects and it should also be noted that the vesicle internalisation defect of *vps13*Δ is not corrected. It was recently documented that endocytic plasma membrane invaginations associate with the cortical ER and this contact facilitates actin polymerisation and endocytic vesicle scission (Encinar del Dedo et al., 2017). Myo5 is a protein that establishes the contact between the ER and endocytic sites by binding Osh2 and Osh3. Osh2/3 in turn bind Scs2 and Scs22, ER membrane proteins, and the Osh2 sterol transfer domain is required for the onset of actin polymerisation at endocytic sites (Encinar del Dedo et al., 2017). Myo3 exhibits a similar domain structure to Myo5 and can possibly perform similar functions at endocytic sites. Previous results are consistent with the view that type I myosin could induce formation of ER-plasma membrane contact sites and this capacity is required for myosin-Osh2/3-Scs2/22 link and the sterol transfer activity of Osh2, actin polymerisation and endocytic vesicle scission (Encinar del Dedo et al., 2017). In the *osh2*Δ *osh3*Δ and *scs2*Δ *scs22*Δ strains, the lifespan of Abp1 patches was increased, with a high fraction of patches initiating the inward movement and retracting back to the plasma membrane (Encinar del Dedo et al., 2017), similar to what we observed for *vps13*Δ. Our results showing that *vps13*Δ defects in endocytosis are suppressed by *MYO3-N* in an Osh2/3-dependent way suggest that one mechanism of suppression could be by promoting the formation of ER-plasma membrane connections at endocytic sites, which involve type I myosin. Also, a recent report suggests that Vps13 could be a binding partner of Osh2 (Bean et al., 2018). This implies a possible Vps13 function in the formation of ER-plasma membrane contact sites, which are important for maintaining the lipid and protein composition of plasma membrane and are a prerequisite for SDS-stress survival. A more efficient action of myosin in promoting sterol transfer by Osh2/3 at endocytic sites helps to overcome the defects caused by the lack of Vps13. This suggests that: Vps13 may be important for the transfer of some lipids to plasma membranes at some ER-lasma membrane contact sites; a lack of Vps13 participation in the formation of contact sites affects endocytosis; and this defect could be overcome by activation of endocytic ER-plasma membrane contact sites. This model is supported by a recent study in which the N-terminal fragment of Vps13 was shown to be able to transfer the bulk of glycerolipids between liposomes (Kumar et al., 2018), implying that Vps13 might relocate lipids between membranes. Such transfer could possibly be required for growth of plasma membrane invagination or vesicle scission during the internalisation step of endocytosis. It requires further study to find out whether the role of Vps13 in endocytosis is direct, since the SDS growth and endocytosis defect of *vps13*Δ may result also from activation of calcium signalling. Since the growth defect of *vps13*Δ on SDS medium is more severe than that of *pmc1*Δ, the activation of calcium signalling is probably not the only reason and other mechanisms may contribute.

In this study, we demonstrate the relationship between Vps13 and calcium signalling, and the importance of Osh2/3-dependent membrane contact sites for bypassing the *vps13*Δ growth defect. Our results will be helpful for studies to better understand the pathogenesis of ChAc in higher eukaryotes and in cell lines. The multicopy suppressor that we selected can suppress the other phenotypes of *vps13*Δ, not just the SDS hypersensitivity. In addition, chemical compounds such as EGTA or FK506 can suppress the SDS hypersensitivity. Thus, we believe that this phenotype is useful to learn about pathways that could be potential targets for chemical intervention, and could be used directly to screen for drugs to treat ChAc.

## MATERIALS AND METHODS

### Strains, media and growth conditions

*Escherichia coli* strain DH5α was used for plasmid propagation. The yeast *S. cerevisiae* strains used in this study are listed in Table S1. For genetic analysis, strains BY4741 *cnb1*Δ *vps13*Δ and BY4741 *crz1*Δ *vps13*Δ were constructed by deletion of *VPS13* using the *vps13::URA3* cassette (pKA475) in the single-deletion strains BY4741 *cnb1*Δ and BY4741 *crz1*Δ. Gene disruptions were performed by transformation of yeast cells with a PCR product containing the *URA3* selection marker flanked by 50 bp of homology to the 5′ and 3′ regions of *VPS13*. The *vps13::URA3* cassette was also used to construct strains encoding Las17-mRFP, Myo3-GFP, Myo5-GFP or Abp1-mCherry endocytic markers in the genome and devoid of Vps13 protein. Genomic integrations were confirmed by PCR on genomic DNA.

Yeast were grown at 30°C in liquid YPD complete medium (1% yeast extract, 2% peptone, 2% glucose) or in synthetic SC medium (0.067% yeast nitrogen base without amino acids, 2% glucose) with desired supplements (adenine, uracil, amino acids) (all media ingredients: DB, Sparks, USA). For growth tests, cells were grown overnight in liquid media and cultures were diluted with water to obtain a cell density equal to OD_600_∼1. Subsequently, aliquots of 4-fold serial dilutions of cells were spotted on plates with solid media. Media used for growth tests were YPD with, or without, addition of SDS (Sigma-Aldrich, St Louis, USA), and SC-leu-arg with, or without, addition of canavanine (Sigma-Aldrich), as described in the figure legends. Plates were incubated at 30°C for 2 (YPD) or 3 (YPD+SDS) days, or as indicated. Mutant cells were very sensitive to small changes in SDS concentration and final SDS concentration in the medium strongly depends on the lot of medium and lot of SDS stock solution, and various physical factors; therefore, each growth experiment was performed using several dilutions of SDS stock solution and a representative result is shown. For two-hybrid analysis, the PJ69-4A strain was used and respective transformants were grown on SC-trp-leu plates, replicated on SC-trp-leu-his supplemented with 1.5 mM aminotriazol (Sigma-Aldrich) and incubated for 3-5 days. Liquid cultures were inoculated with a mix of several yeast colonies obtained after transformation. Growth tests were repeated at least twice. Plates were scanned by HP Scanjet G4010 or Epson Perfection 2480 Photo scanner. Imagines were processed with Adobe Photoshop 6.0.

### Plasmids

Plasmids used are described in Table S2. To subclone the *MYO3* gene fragment, the *Bam*HI and *Sal*I DNA fragment from the original clone from the genomic bank was transferred into pRS425-P_GPD_ vector to obtain pRS425-P_GPD_-MYO3-N. Further transferring a 3.2 kb *Bam*HI *Hin*dIII fragment of pRS425-P_GPD_-MYO3-N to pRS425-P_GPD_ generated pRS425-P_GPD_-MYO3*-*NΔ680-775*.* To destroy calmodulin-binding motifs IQ1 and IQ2 in Myo3-N, mutations were introduced in the *MYO3-N* fragment, after subcloning into pUC19 using *Pst*I and *Sal*I enzymes, by one-step site-directed mutagenesis. Three plasmids were obtained: pUC19-MYO3-iq1, encoding myosin fragment with I725G and Q726G substitutions; pUC19-MYO3-iq2, encoding protein with I743G and Q744G substitutions; and pUC19-MYO3-iq1/2, encoding protein with all four substitutions. Next, the *Pst*I-*Sal*I fragments were transferred back to create pRS425-P_GPD_-myo3-iq1, pRS425-P_GPD_-myo3-iq2 and pRS425-P_GPD_-myo3-iq1/2. pRS425-P_GPD_-MYO3-N-HA and pRS425-P_GPD_-myo3-iq1/2-HA were generated by introducing the HA tag into the *Sal*I polylinker restriction site. Sequences of oligonucleotides used in this study are available upon request.

To construct plasmids for two-hybrid systems, the *CMD1* gene and mutated alleles were amplified by PCR using YEplac181lac bearing *CMD1*, *cmd1-226*, *cmd1-228*, *cmd1-231* or *cmd1-239* alleles as the templates. PCR products were digested with *Pst*I and *Bam*HI and were cloned into pGAD424. To obtain pGBT9-MYO3-N or pGBT9-myo3-iq1/2, the appropriate fragments were amplified by PCR with pRS425-P_GPD_-MYO3-N or pRS425-P_GPD_-myo3-iq1/2 as the templates. PCR products were cloned into *Eco*RI and *Sal*I sites of pGBT9.

### Screen for multicopy suppressors of *vps13-I2749R* mutation

The *vps13*Δ [*vps13-I2749R*] cells were transformed with pFL44-based genomic bank (Kabir et al., 2005), plated on SC-ura-leu plates and incubated at 30°C for 3 days. Then, transformants were replicated on YPD plates with addition of 0.03% SDS and incubated at 30°C for 3 days. Positive clones were isolated and grown in SC-ura liquid medium for plasmid isolation. Plasmids were retransformed and suppression of the *vps13*Δ [*vps13-I2749R*] growth defect was confirmed.

### Actin staining and fluorescence microscopy

For actin staining, cells were grown in SC-leu medium to log-phase, fixed with formaldehyde (Sigma-Aldrich for 2 h, washed and stained with Alexa-Fluor-546-conjugated phalloidin (Thermo Fisher Scientific, Waltham, USA). Cells were viewed with an Eclipse E800 (Nikon, Tokyo, Japan) fluorescence microscope equipped with a DS-5Mc camera (Nikon, Tokyo, Japan). Images were collected using Lucia General 5.1 software (Laboratory Imaging Ltd, Prague, Czech Republic). The same fields were viewed by differential interference contrast (DIC) optics. All samples in the experiment were encoded and the microscope observations and quantifications were performed blindly. Charts and statistical analyses were done using Microsoft Excel.

For live-cell imaging, cells expressing tagged proteins were visualised after growing to early log-phase in synthetic medium with appropriate supplements. Epifluorescence microscopy was performed using Olympus IX-81 inverted microscope (Olympus, Tokyo, Japan) with a DeltaVision RT Restoration Microscopy System (using a 100×/1.40 NA oil objective) and Photometrics Coolsnap HQ camera, with imaging and image capture performed using SoftWoRx™ image analysis and model-building application (Applied Precision Instruments, Seattle, USA). Live-cell imaging of GFP-tagged Las17 and mCherry-tagged Abp1 proteins was performed with 1 s time lapse. All image data sets were deconvolved, using the SoftWoRx application. Live-cell images of Myo3-GFP and Myo5-GFP were acquired using OMX DeltaVision V4 microscope (GE Healthcare Life Sciences, Chicago, USA) and a 60× USPLAPO (1.42 NA) objective with refractive index 0.1514 immersion oil (Cargille, Cedar Grove, USA) with 0.5 s time lapse. Samples were illuminated using Insight Solid State Illuminator (10%), and images were taken simultaneously on separate scientific complementary metal oxide semiconductor (sCMOS) cameras (30 ms exposure). Seven 250 nm sections were acquired every 500 ms (181 time points). The stacks were then deconvolved and processed, using SoftWorx, to produce a movie composed of maximum intensity projections at each time point. The Myo3-/Myo5-GFP lifetime was analysed from those projections. Movies and kymographs were assembled using Fiji software. Charts and statistical analyses were done in GraphPad Prism (https://www.graphpad.com/scientific-software/prism/, 02.08.2018).

### Western blot analysis

Yeast cells were grown at 30°C in SC-ura-leu to the log-phase. Protein extracts were prepared after disrupting cells with glass beads as described (Rzepnikowska et al., 2017a) or alkaline lysis (Yaffe and Schatz, 1984). Samples were analysed by standard SDS-PAGE followed by western blotting using mouse monoclonal anti-Pgk1 (dilution 1:20,000, Molecular Probes Cat# A-6457, RRID:AB_221541, Thermo Fisher Scientific), monoclonal anti-HA epitope (dilution 1:2000, Cat# 901503, BioLegend, San Diego, USA), rabbit anti-phospho-p44/42 MAPK (Erk1/2) (Thr202/Thr204; dilution 1:1000, Antibody #9101, Cell Signalling Technology, Danvers, USA; validation: Pérez et al., 2016), recognising phospho-Slt2, antibodies. Secondary anti-mouse or anti-rabbit IgG horseradish peroxidase (HRP)-conjugated antibodies (dilution 1:2000, Dako, Glostrup, Denmark) were also used. Signal was detected by enhanced chemiluminescence (Millipore, Darmstadt, Germany). Densitometry was performed with ImageJ (https://imagej.nih.gov/ij/, 02.08.2018). Chart and statistical analyses were done in GraphPad Prism.

### β-galactosidase activity

For the β-galactosidase activity assay, cells were grown overnight in SC-ura or SC-ura-leu, collected and extracts prepared with glass beads. Activity of β-galactosidase was analysed as described (Kamińska et al., 2005). In each of three experiments, at least three independent transformants of respective yeast strains were inoculated, and their extracts were assayed in duplicates.

## Supplementary Material

Supplementary information
