# Supplementary material for: Yeast-model-based study identified myosin- and calcium-dependent calmodulin signalling as a potential target for drug intervention in chorea-acanthocytosis
Source: Dis Model Mech. 2019 Jan 28;12(1):dmm036830. doi: 10.1242/dmm.036830 (PMC6361151; doi:10.1242/dmm.036830)
Supplement: Supplementary information [file dmm-12-036830-s1.pdf]

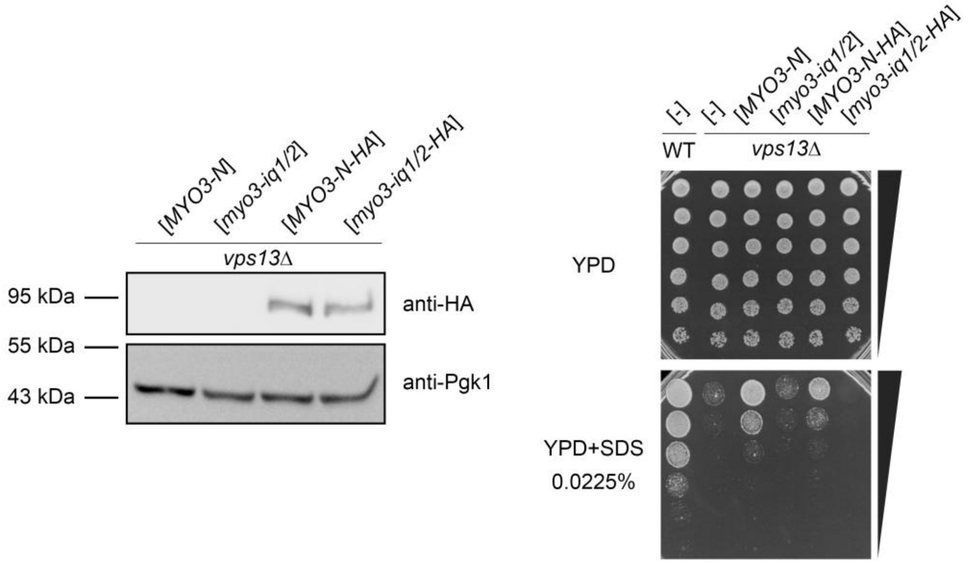

Fig. S1. *MYO3-N-HA* and *myo3-iq1/2-HA* are similarly expressed and similarly influence *vps13Δ* growth on SDS plates as alleles without the tag. Western blot analysis of yeast extracts from strains indicated (left panel) and growth of respective strains on SDS plates (right panel). Cells were grown to early exponential phase, cell suspensions were diluted and dropped on plates. The extracts were prepared by alkaline lysis.

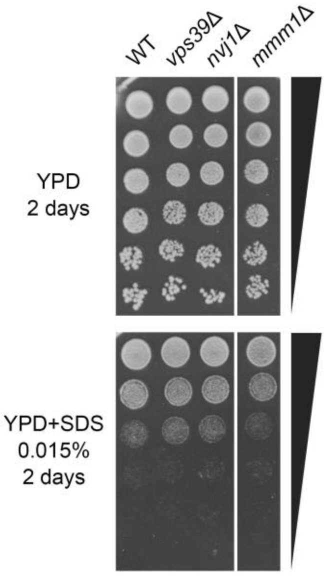

Fig. S2. Mutants showing defects in various membrane contact sites are not hypersensitive to SDS. Growth test of indicated mutants was performed. Irrelevant line was removed.

Table S1. ***Saccharomyces cerevisiae*** **strains used.**

**Strain**	**Genotype**	**Source or reference**
BY4741	*MAT***a**, *his3Δ1, leu2Δ0 met15Δ0 ura3Δ0*	Open Biosystem
BY4742	*MATα, his3Δ1, leu2Δ0 met15Δ0 ura3Δ0*	Open Biosystem
BY vps13Δ	*MAT***a** *his3Δ1 leu2Δ0 met15Δ0 ura3Δ0 vps13::kanMX*	Open Biosystem
BY crz1Δ	*MAT***a** *his3Δ1 leu2Δ0 met15Δ0 ura3Δ0 crz1::kanMX*	Open Biosystem
PS3	*MAT***a** *his3Δ1 leu2Δ0 met15Δ0 ura3Δ0 cnb1::kanMX*	This study
WR11	*MATα LAS17-7Ala–mRFP::HIS3 ura3-52 leu2-3,112 his3Δ200 trp1-1 lys2-801 vps13::URA3*	This study
KAY1466	*MATα his3Δ1 leu2Δ0 lys2Δ0 ura3Δ0 ABP1-mCherry::HIS3*	Palmer et al. 2015
KAY1931	*MATα his3Δ1 leu2Δ0 lys2Δ0 ura3Δ0 ABP1-mCherry::HIS3 vps13::URA3*	This study
KAY757	*Mat a his3Δ1 leu2Δ ura3Δ met15Δ LAS17-GFP::HIS3*	Invitrogen
KAY1930	*Mat a his3Δ1 leu2Δ ura3Δ met15Δ LAS17-GFP::HIS3 vps13::URA3*	This study
KAY758	*Mat a his3Δ1 leu2Δ ura3Δ met15Δ MYO3-GFP::HIS3*	Invitrogen
KAY1939	*Mat a his3Δ1 leu2Δ ura3Δ met15Δ MYO3-GFP::HIS3 vps13::URA3*	This study
KAY1940	*Mat a his3Δ1 leu2Δ ura3Δ met15Δ MYO5-GFP::HIS3*	This study
KAY1941	*Mat a his3Δ1 leu2Δ ura3Δ met15Δ MYO5-GFP::HIS3 vps13::URA3*	This study
KJK177	*MAT***a** *his3Δ1 leu2Δ ura3Δ met15Δ crz1::kanMX vps13::URA3*	This study
PS4	*MAT***a** *his3Δ1 leu2Δ ura3Δ met15Δ cnb1::kanMX vps13::URA3*	This study
pJ69-4a	*MAT***a** *trp1Δ-901 leu2-3112 ura3-52 his3-Δ200 gal4 Δ gal80Δ GAL2-ADE2 LYS2::GAL1-HIS3 met2::GAL7-lacZ*	James et al., 1996
SCMIG1502	*MATα his3Δ leu2Δ lys2Δ ura3Δ osh2Δ::kanMX osh3Δ::KMX*	Encinar del Dedo et al., 2017
KJK188	*MATα his3Δ leu2Δ lys2Δ ura3Δ osh2Δ::kanMX osh3Δ::KMX vps13::URA3*	This study
BY ede1Δ	*MAT***a** *his3Δ1 leu2Δ0 met15Δ0 ura3Δ0 ede1Δ::kanMX*	Open Biosystems
BY sla1Δ	*MAT***a** *his3Δ1 leu2Δ0 met15Δ0 ura3Δ0 sla1Δ::kanMX*	Open Biosystems
KAY1482	*MATα his3Δ1 leu2Δ0 lys2Δ ura3Δ0 rvs167Δ::LEU2 GFP-Snc1-SUC2 URA3*	Smaczynska-de Rooij et al., 2012
BY end3Δ	*MATα, his3Δ1, leu2Δ0 met15Δ0 ura3Δ0 end3Δ::kanMX*	Open Biosystem
BY sla2Δ	*MATα, his3Δ1, leu2Δ0 met15Δ0 ura3Δ0 sla2Δ::kanMX*	Open Biosystem
BY cog5Δ	*MATα, his3Δ1, leu2Δ0 met15Δ0 ura3Δ0 cog5Δ::kanMX*	Open Biosystem
BY cog8Δ	*MATα, his3Δ1, leu2Δ0 met15Δ0 ura3Δ0 cog8Δ::kanMX*	Open Biosystem
BY vps15Δ	*MATα, his3Δ1, leu2Δ0 met15Δ0 ura3Δ0 vps15Δ::kanMX*	Open Biosystem
BY vps17Δ	*MATα, his3Δ1, leu2Δ0 met15Δ0 ura3Δ0 vps17Δ::kanMX*	Open Biosystem
BY vps35Δ	*MATα, his3Δ1, leu2Δ0 met15Δ0 ura3Δ0 vps35Δ::kanMX*	Open Biosystem
BY vps27Δ	*MATα, his3Δ1, leu2Δ0 met15Δ0 ura3Δ0 vps27Δ::kanMX*	Open Biosystem

Disease Models & Mechanisms • Supplementary information

BY stp22Δ	*MATα, his3Δ1, leu2Δ0 met15Δ0 ura3Δ0 stp22Δ::kanMX*	Open Biosystem
BY vps36Δ	*MATα, his3Δ1, leu2Δ0 met15Δ0 ura3Δ0 vps36Δ::kanMX*	Open Biosystem
BY vps24Δ	*MATα, his3Δ1, leu2Δ0 met15Δ0 ura3Δ0 vps24Δ::kanMX*	Open Biosystem
BY pmc1Δ	*MATα, his3Δ1, leu2Δ0 met15Δ0 ura3Δ0 pmc1Δ::kanMX*	Open Biosystem
BY vps39Δ	*MATα, his3Δ1, leu2Δ0 met15Δ0 ura3Δ0 vps39Δ::kanMX*	Open Biosystem
BY nvj1Δ	*MATα, his3Δ1, leu2Δ0 met15Δ0 ura3Δ0 nvj1Δ::kanMX*	Open Biosystem
BY mmm1Δ	*MATα, his3Δ1, leu2Δ0 met15Δ0 ura3Δ0 mmm1Δ::kanMX*	Open Biosystem

Table S2. List of plasmids used.

**Plasmid**	**Source or reference**
pRS415	Sikorski and Hieter, 1989
pRS415-VPS13	Rzepnikowska et al., 2017a
pRS415-vps13-I2749R	Rzepnikowska et al., 2017a
pRS425-P_{GPD}	Sikorski and Hieter, 1989
pRS425-P_{GPD}-MYO3-N	This study
pRS425-P_{GPD}-MYO3-N(Δ680-775)	This study
pRS425-P_{GPD}-myo3-N-iq1	This study
pRS425-P_{GPD}-myo3-N-iq2	This study
pRS425-P_{GPD}-myo3-N-iq1/2	This study
pYEp181lac	Gietz and Sugino, 1988
pYEp181lac-CMD1	This study
pYEp181lac-cmd1-226	Schaerer-Brodbeck and Riezman, 2003
pYEp181lac-cmd1-228	Schaerer-Brodbeck and Riezman, 2003
pYEp181lac-cmd1-231	Schaerer-Brodbeck and Riezman, 2003
pYEp181lac-cmd1-239	Schaerer-Brodbeck and Riezman, 2003
pAMS363 (CDRE-*LacZ*)	Stathopoulos and Cyert, 1997
pGBT9	Clontech
pGBT9-MYO3-N	This study
pGBT9-myo3-iq1/2	This study
pGAD424	Clontech
pGAD424-CMD1	This study
pGAD424-cmd1-226	This study
pGAD424-cmd1-228	This study
pGAD424-cmd1-231	This study
pGAD424-cmd1-239	This study
pKA475 (*URA3* cassette)	Smaczynska-de Rooij et al., 2008
pRK107S (*PMC1*)	Kucharczyk et al., 2000
pRK109S (*PMR1*)	Kucharczyk et al., 2000
pRS425-P_{GPD}-MYO3-N-HA	This study
pRS425-P_{GPD}-myo3-N-iq1/2-HA	This study

B. Kymographs of Las17-GFP, Myo3-GFP and Myo5-GFP patches. Two representative kymographs for each strain, as in A, are shown. Las17-GFP patches were observed for 120 s using an Olympus IX-81 microscope and imaging was performed via 1 s time-lapse. Myo3-GFP and Myo5-GFP were observed for 90 s using an OMX DeltaVision V4 microscope and imaging was performed via 0.5 s time-lapse.
C. Kymographs of Abp1-mCherry patches. Two representative kymographs for each class of patch behavior are shown. Abp1-mCherry patches were viewed for 90 s using an Olympus IX-81 microscope and imaging was performed via 1 s time-lapse. Quantification of the patches was analysed using a two-tailed Student t-test (n=5, patches counted

Disease Models & Mechanisms • Supplementary information
